# Sex-Specific Behavioral Features of the Prenatal Valproic Acid Rat Model of Autism Spectrum Disorder

**DOI:** 10.3390/brainsci15040388

**Published:** 2025-04-09

**Authors:** Patience Mulalo Mamali, Christine Dignon, Ayanda Ngwenya, Busisiwe Constance Maseko

**Affiliations:** School of Anatomical Sciences, University of the Witwatersrand, Parktown, Johannesburg 2193, South Africa; patience.mamali2@wits.ac.za (P.M.M.); 711602@students.wits.ac.za (C.D.); ayanda.ngwenya@wits.ac.za (A.N.)

**Keywords:** autism model, valproic acid, anxiety, repetitive behavior, sociability

## Abstract

**Background/Objectives:** Autism is a complex neurodevelopmental disorder characterized by restricted behaviors and impaired social and communication skills. The exact cause of autism remains unknown. One promising animal model for studying autism is the valproic acid rat model. Due to a 1 to 4 bias for males in autism occurrence, most animal model studies investigate only males and neglect females. However, female autism often appears different from that observed in males. Females are said to be less regularly diagnosed because they can “mask” their symptoms. Female autism is as necessary to investigate as male autism. **Methods:** Fertile adult female Sprague-Dawley rats were impregnated and injected with valproic acid on gestational day 13. Male and female offspring were subjected to behavioral tests to investigate autistic symptoms. Tests included novel object recognition, balance-beam, Y-maze, hole-board, three-chamber, marble burying, olfactory, light/dark and hot plate tests. **Results:** The tests revealed that VPA-exposed rats had increased anxiety-like behaviors, hyperactivity, and impaired non-verbal communication. However, they did not display repetitive behaviors or cognitive impairments. Notably, male and female rats showed different autism-like traits, with both showing hyperactivity, and males (but not females) additionally showing impaired sociability and increased anxiety. **Conclusions:** The findings suggest that prenatal exposure to VPA induces autism-like behaviors in both male and female Sprague-Dawley rat offspring. However, males appear more impacted by VPA exposure as evinced by their display of more autism-like symptoms relative to females. This study provides support for including both sexes in all studies modelling autism, as outcomes are seemingly impacted by the sex being observed.

## 1. Introduction

Autism Spectrum Disorder (ASD) has become the most commonly diagnosed neurodevelopmental disorder, with its prevalence increasing rapidly [1,2]. This increase has been seen as a result of complex combinations of immunological, environmental, and neurological factors, rather than that of increased awareness or improved diagnostics [3,4]. However, this claim is controversial, as some authors assert that increased awareness and diagnostic technologies are the main drivers of perceived increasing prevalence [5]. Most likely, the true rationale is likely a combination of all these factors; for example, as emerging economies develop more research and diagnostic resources, their prevalence of ASD will appear to increase, as more diagnoses will be made.

Autism is defined in the fifth edition of the Diagnostic Statistical Manual (DSM-V TR) [6] as characterized by “persistent deficits in social communication and interaction across multiple contexts (reciprocation, non-verbal communication and relationships), and the presence of restrictive, repetitive patterns of behavior, interests or activities”. Autism can also be associated with maladaptive behaviors such as hyperactivity, anxiety, irritability/mood instability (aggression and temper outbursts), and self-injury [7]. The condition can develop before the age of 36 months and has a tendency to occur more often in males than in females, with a ratio of 4:1 [8,9]. As of 2022, the worldwide prevalence of ASD is estimated to be 0.6%, with the highest prevalence reported in Australia at 1.7% [10]. The investigators assert that such deviations in regional prevalence is due to different mechanisms of diagnosis and even definitions of what ASD is [10]. Between the ages of 18 to 24 months, there is often a regression of 30% in developmental skills [9], because of abnormal brain development causing developmental delays [11]. Increasingly, evidence is emerging that points to males and females with ASD having neurobiological differences with respect to neuroanatomy, neuroimmunology, and neurogenetics [12,13]. For example, females are reported to have relatively reduced cerebellar gray matter volumes and fibers projecting between the corpus callosum and frontal cortex [13], while their temporal cortex and white matter fibers are markedly increased relative to males with autism, as well as neurotypical males and females [12,13]. When considering neuroimmunology, greater numbers of activated microglia have been observed in the cerebellum in ASD males [12], while in studies that investigate neurogenetics of ASD, most genes identified as associated with ASD have been reported to be expressed disproportionately more in males than females [12].

Currently, there are several hypotheses as to the etiology of autism. Although most are commonly linked to genetics [14], exposure to teratogens during fetal development, such as thalidomide [15], ethanol [16], and valproic acid (VPA) [16,17,18], has been reported as leading to the development of ASD. Valproic acid, an anticonvulsant drug, is widely used as a treatment for epilepsy, mood instabilities, and migraines, and was first introduced in France in 1964 and in the US in 1978 [19]. When used for treatment in pregnant women, the drug was found to have increased the risk of autism development significantly, especially when exposed during the first trimester [17,20,21]. Therefore, to elucidate the underlying neurobiological mechanisms of autism, VPA-exposed animal models have been developed to capture major symptoms and allow for the rigorous study of molecular, cellular, and behavioral alterations undergirding ASD [22,23].

Multiple studies using VPA administered in early embryogenesis and before completion of neural tube closure in rats established striking similarities of behavior and anatomy between the VPA rat model and autism [24,25,26,27], which are useful in modeling the condition despite the inherent limitation that animal models can only to an extent truly mimic the genetic and environmental complexity of ASD. These studies have many similarities in the methodological design of their behavioral tests; however, variations include (i) the sex, (ii) sample sizes, and (iii) the variation in the number and types of behavioral tests employed. The tests conducted vary according to the underlying aim of the research, but many studies tend to utilize fewer behavioral tests. A common feature of most studies in this field is the exclusion of female animals in the analysis, introducing a significant additional limitation in the field [28,29,30,31], as males and females with ASD are increasingly shown to possess distinct sex-specific differences in their neuroanatomy, neuroimmunology, and neurogenetics [12]. As such, there is a shortage in descriptions of the typical appearance of autism-like behaviors in female rat models. This study sought to conduct a comprehensive battery of tests to evaluate behavioral outcomes in the VPA-induced model of autism when described in an inclusive cohort of both males and females.

## 2. Materials and Methods

### 2.1. Animals

**Figure 1:**
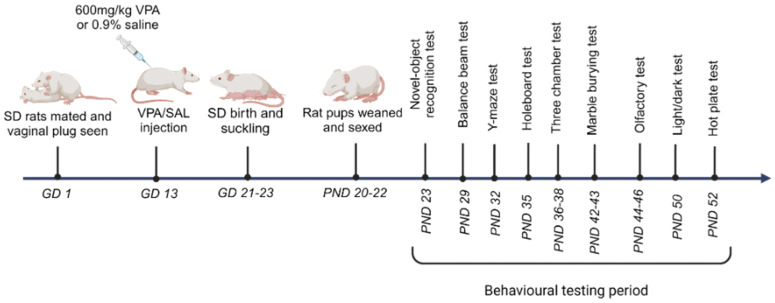
Illustrating the schedule of animal impregnation and treatment to induce prenatal valproic acid exposure in pups; and subsequent sequence and timeline of behavioral testing. SD—Sprague-Dawley rats, VPA—valproic acid, SAL—0.9% saline solution, GD—gestational day, PND—postnatal day.

The breeding, treatment, and testing sequence of the currently used animals is depicted in Figure 1. Fertile adult nulliparous female Sprague-Dawley (SD) rats (Rattus norvegicus, 10–12 weeks old) acquired from the Wits Research Animal Facility (WRAF) (University of the Witwatersrand, Johannesburg, South Africa) were mated naturally overnight in metabolic cages. After confirmation of mating (based on fallen vaginal plugs), the dams were separated from their breeding mates. We randomly assigned pregnant rats into two groups, namely, valproic acid (VPA)-exposed (*n* = 4) and saline (SAL)-injected (or control) groups (*n* = 4). The VPA group received a single subcutaneous injection of valproic acid sodium salt (Sigma-Aldrich, St. Louis, MO, USA) dissolved in isotonic sterile saline on gestational day 13 (GD 13) per individual body weight at 600 mg/kg, while the control group received a single subcutaneous injection of isotonic saline on the same day (adapted from a previous study [32]). The pregnant rats were housed in enriched individual cages until births occurred.

The cages were constructed out of Perspex plastic and ventilated, with the following dimensions: 41 × 28 × 15.3 cm, with floors covered with wood chips and shredded paper as bedding. A 12 h light/dark cycle (06h00–18h00) was maintained, and standard rat chow and water were available ad libitum. The pregnant rats were maintained at a room temperature of 25 ± 2 °C and monitored by weighing on GD one and every three days thereafter, as well as by general observation daily. Births occurred naturally on GD 20 to 23.

Pups suckled for 20 to 22 days and then naturally weaned, at which stage they were sexed and separated from dams. Male (*n* = 20) and female (*n* = 23) pups from the control group and male (*n* = 15) and female (*n* = 17) pups from the VPA group were obtained. The pups were then placed in individual cages and allowed to acclimatize with free access to food and water. The pups were acclimatized for two days post separation from their mothers, in preparation for behavioral tests. The pups were handled to acclimatize them to behavioral training and tests, which commenced from postnatal day 23 (PND23). The age for each behavioral test was guided by previous studies from which all tests were adopted, and tests were staggered to allow individuals to rest between tests to minimize stress. All but the marble burying test (photographed) were video recorded using the Logitech recording system (C270 USB HD Webcam, Logitech, Lausanne, Switzerland) and tracked via AnyMaze software (version 7.1, Stoelting Co., Wood Dale, IL, USA).

### 2.2. Behavioral Tests

1. Response to social stimulus/sociability: three-chamber sociability test

The three-chamber sociability test was used to assess sociability [18,33]. This behavioral test was run on three consecutive days on approximately 38-day-old SD rats. The apparatus was constructed from a black rectangular box made of plexiglass (101 × 101 × 20 cm). It was partitioned into three equal compartments or chambers by two plexiglass partitions, each with a removable door leading to the center chamber. In one distal chamber, the novel rats were habituated by placing them under a weighted and perforated inverted metal cup (17.8 cm diameter). To avoid stressing the participant rats, the novel rats were habituated a day ahead of testing day to ensure docile behavior consistent with minimum signs of stress. This was performed under observation, twice for 15 min per rat. Any rats who behaved erratically or showed signs of stress were excluded from the test. Novel rats are those that were designated as social objects rather than test participants in the test and had been kept in separate home cages.

All the subject rats were acclimatized for one hour in the test room before the sociability assessment. The three-chamber test consisted of three 10 min sessions. The first session allowed for the participant rat to acclimatize to the apparatus, only within the center chamber. The second session allowed for the participant rat to acclimatize to the entire apparatus, moving freely within all three empty chambers. The final session included a novel rat under an inverted wire cup in the right chamber (“social chamber”) and a novel object under the inverted cup in the left chamber (“non-social chamber”) and allowing the subject rat to explore all three chambers.

Parameters assessed were number of entries into the social and non-social chambers, time spent in each of the three chambers, and finally, a sociability index was calculated according to that of Baronio et al. [34]:

Sociability Index = (time in social chamber − time in nonsocial chamber)/(time in social chamber + time in nonsocial chamber).

2. Non-verbal communication: cotton-tip based olfactory test

We used the cotton-tip based olfactory habituation/dishabituation test to assess olfaction in SD rats. Rats mostly communicate socially by emitting and detecting olfactory pheromones [33]. Social interaction can be enhanced using olfactory cues like maternal scent and pheromones from urine of the opposite sex [27,33,35]. Duration of scent sniffing time and recognizing the olfactory stimuli of familiar vs novel odors was used to test olfactory communication [18,33]. The stepwise decreasing interaction is a natural response in rats as they become accustomed to an odor [36], a behavior used to indicate habituation ability. In contrast, dishabituation is described as the sudden increase in sniffing time when a new odor is presented [36]. Inability to dishabituate shows a lack of olfactory memory, compromised non-verbal communication, and inability to selectively focus/discriminate salient from non-salient stimuli [33,37].

This test was conducted over 2 days on approximately 48-day-old rats. A cotton tip with odorant was placed at the center of the testing apparatus, a field of 53 × 44 × 27 cm dimensions. Water, lemon extract, and urine from the opposite sex were used as control, non-social, and social scents, respectively. The urine had been collected from 46-day-old male and female SD rats. The rats were acclimatized for 5 min in the testing room prior to testing. To clear the testing room of any odors in between scent presentations, it was ventilated for 1 min using a fan.

The test was composed of three trials per scent per rat such that water (water 1–3), lemon extract (lemon extract 1–3), and urine (urine 1–3) were presented three times each. Each trial was 2 min with 1 min inter-trial intervals. Odor detection was defined as a nose sniff within 2 cm of the odorant [37,38], of which low sniffing durations represented familiarity with odors [36,38]. This protocol was adopted from previous protocols [18,35,39].

3. Repetitive/restrictive behavior, lack of interest, and anxiety-like behaviors: hole board, marble burying, and light/dark test

The hole board, marble burying, and light/dark tests were employed to assess lack of interest (or reduced exploratory behavior), repetitive behavior, and anxiety-like behaviors, respectively. Repetitive behavior such as excessive handwashing, repetitive rocking and tapping are observed in humans with autism; similarly, self-grooming or digging is observed in autism rat models [40]. Exploratory behavior was observed through the hole board, while repetitive behavior was tested using marble burying (MB) and anxiety through the light/dark test.

(a) Hole Board Test

The hole board test was used to evaluate the willingness, or lack thereof, to explore an unknown environment. This behavioral test was completed in one day on 35-day-old rats, using a plexiglass box with black walls (53 × 44 × 27 cm) and a black wooden floor. The apparatus had a total of sixteen holes in the base, in a 4 × 4 arrangement, placing four holes at the center and twelve at the periphery, all equidistant from each other. Food pellets (peanut butter rolled in seeds) were placed in the four holes in a diagonal arrangement that included two peripheral and two central holes. This was to incentivize and encourage exploration. The rats were acclimatized for 1 h in the testing room prior to the test. They were then placed at the center of the hole board and allowed to explore the apparatus for 5 min whilst being video recorded.

(b) Marble Burying Test

Research shows that rodents engage in the action of burying in response to a non-aversive object such as marbles or food pellets, which accurately reflects repetitive digging, behavior that is highly change resistant and perseverative [41]. The marbles were washed with mild laboratory detergent and rinsed in tap water and dried prior to each use and wiped with 70% ethanol in between trials [40]. The marble burying test was conducted over two days in 42–43-day-old rats. Twelve colorful marbles were used, each 35 mm in diameter and weighing 53 g. The marbles were arranged in a 3 × 4 arrangement in the participants’ home cage, with newly changed sawdust bedding filled to 6 cm thickness [41]. Prior to each test, the rats were acclimatized in the testing room for 1 h and then acclimatized in the new cage with fresh bedding for 5 min prior to addition of marbles. The rat pups were then placed back in the testing room for 20 min, and a picture was taken for scoring later. The marble burying test involved rats displacing the bedding to bury the glass marbles, and a marble was considered buried if 2/3 of it was no longer visible from above the bedding [41].

(c) Light/Dark Test

The light/dark test is performed in a two-compartment apparatus, in which one chamber is enclosed and dark whilst the other chamber is open and brightly lit [33]. The literature has highlighted that anxiety-prone rats show reduced exploration tendencies and prefer enclosed and dark compartments, whilst comfortable rats tend to explore by spending more time in the open and lit compartment in the light/dark apparatus [33,42]. We performed the light/dark test in one day (adopted from the previous literature [43,44]), on 50-day-old rats. A black plexiglass apparatus (with one transparent wall, 37.5 × 37.5 × 41.5 cm) was divided into two equal halves, one enclosed and dark chamber, and the other transparent and lit. The dark and light compartments were divided by a black wall, connected with an opening covered by a removable door. We acclimatized the subject rats for 30–35 min in the testing room before commencing with the test. Each participant rat was placed in the dark compartment for 3 s, after which the door was removed, and the rat was allowed to freely explore between the light and dark chambers. The test was video recorded for 10 min for later analysis. The time spent in each compartment was then used to calculate an anxiety index, which in this study is defined as follows:

Anxiety index = (time in dark zone)/(time in dark zone + time in light zone).

In addition, the amount of time spent immobile (or freezing) was quantified for each animal, defined as the length of time (s) spent without moving 80% of the body. Finally, the number of entries into the light zone, and the speed of travel, were quantified to assess locomotor activity while undergoing the test.

4. Motor and sensory stimuli: balance beam and hot plate thermal test

The balance beam and the hot plate thermal test were used to assess motor capability and sensory stimuli, respectively [18,33].

(a) Balance Beam Test

This test was conducted on approximately 29-day-old rats. The balance beam apparatus consisted of a square-shaped beam 60 cm long, suspended 50 cm above a table and connecting a flat starting platform (7 cm wide and 20 cm long) and at the end, a wooden escape box (21.5 × 18 × 10 cm). The escape box contained home cage bedding for the specific rat along with a food incentive (peanut or seed). We used a light above the start platform to serve as an aversive stimulus.

To train the animals, the width of the beam was gradually reduced from 4 cm (day 1) to 3 cm (day 2) and ultimately 1.5 cm for the testing day. Each day of training involved three opportunities for each participant animal to practice traversing the beam. On test day, each animal was subjected to four attempts of the test, which were then averaged. An inter-trial interval of 10 min was allowed between each attempt. This protocol was adopted from [45].

(b) Hot Plate Thermal Test

The hot plate thermal test was used to analyze how rats sense and react to mildly painful thermal stimuli [46]. This test is analogous to the human removal of a hand from a heat source, and it is a conscious natural response to thermal pain [46]. The rat typically and reflexively would remove and lick paws, stamp, jump, and do a leaning posture in response to acute heat (50–55 °C) after a few seconds, indicating that the heat on the plate is uncomfortable [46,47].

The hot plate thermal test was conducted in one day on 52-day-old rats. We used a square hot plate (19 × 19 cm width), surrounded by a metal mesh enclosure (15 cm height). The rats were acclimatized to the testing room for 30 min prior to testing on the hot plate. We used a temperature range of 54.5 to 55.5 °C, which was monitored using an infrared thermometer (DT8380AH, Dynamic Instrumentation, Sandton, South Africa). A stopwatch was used to determine the time taken for each rat to respond to the heat stimulus, noted by the reaction to lick hind paws. Rats were then immediately removed from the hot plate. However, if no reaction was observed, rats were removed at 30 s to avoid injury [46,47].

5. Cognition: Novel object recognition and Y-maze tests

(a) Novel Object Recognition Test

This test was used to measure short term memory, which was noted as the time the participant rat spent interacting with the novel object relative to the familiar object.

This test was conducted over three consecutive days on approximately 23-day-old rats. The test arena was demarcated using white tape into a 4 × 4 arrangement of equal partitions, such that 16 square sections were visible. We used a plexiglass box (37.5 × 37.5 × 41.5 cm). On the first day, rats were trained in an empty field, and on the second day, two identical objects were added for familiarization at diagonal corners of the arena. On the third and testing day, rats were first exposed to the two identical familiar objects placed at the same corners as previously, and following a 15 min interval, one familiar object was replaced with a novel object. Each training session was conducted over 5 min, while the test was performed over 3 min. This protocol was adopted from [48]. To assess short term memory, we calculated a discrimination index for each group as follows (adopted from Antunes and Biala [49]):

Discrimination index = (time exploring novel object)/(time exploring novel object + time exploring familiar object).

When the index falls above 0.5 (or 50%), then the group has spent more time with the novel object, indicating recognition of novelty, and thus a discriminative ability [49]. Being within a certain proximity to either the novel or familiar object (2 cm), with the head oriented towards the object, was considered as time exploring the respective object.

(b) Y-Maze Test

This behavioral test was conducted on 32-day-old rats. We used a plexiglass Y-maze with three arms, extended at 120 degrees from each other (each 30 × 9 × 15 cm). The rats were acclimatized for 30 min in the testing room before testing on the Y-maze and were not exposed to the testing apparatus prior to commencement. The rats were tested for 8 min each, by placement at the center of the Y-maze, and allowed to freely move among the arms whilst being recorded. This test is used to determine spontaneous alternations where a decrease in the percentage of spontaneous alternations was taken as an indicator of increased sameness. The series of arm entries were analyzed using AnyMaze software (version 7.1, Stoelting Co., Wood Dale, IL, USA) to determine the number of spontaneous alternations, an alternation occurring when the animal entered the arms in succession. The percentage of spontaneous alternations was calculated using the following equation (adapted from Kumar and Sharma [50]):

%Spontaneous alternations = [(Total alternations)/(Total arms entered − 2)] × 100.

### 2.3. Data Analysis

All statistical analyses in the current study were conducted using GraphPad Prism 8.0.2 and STATA version 17.0 software packages. To test for normality, the Shapiro–Wilk test was conducted. In parametric data, multiple group comparisons were performed through two-way ANOVA, while for non-parametric data, multiple comparisons were conducted through Friedman’s test to identify VPA exposure or sex effects, or interaction between the two factors. If a significant difference was found in any of the factors, Tukey’s or Dunn’s post hoc test was conducted following two-way ANOVA or Friedman’s test, respectively. For intra-group comparisons, we used the student’s *t*-test. For significance, a *p*-value of 0.05 or lower was considered the threshold (*p* ≤ 0.05). Data were expressed as the mean ± standard deviation (SD).

## 3. Results

### 3.1. Valproic Acid Effects on Sociability

#### Three-Chamber Sociability Test

This test was conducted to assess the sociability of VPA-exposed rats compared to non-exposed counterparts. Sociability was indicated by heightened affinity towards the chamber containing a fellow rat as opposed to the center chamber or that containing an inanimate object.

**Figure 2:**
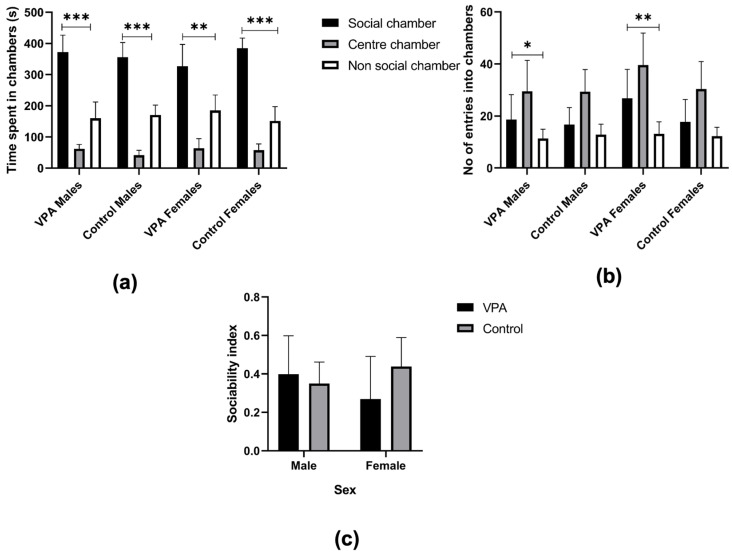
Sociability. Graphs showing outcomes from the three-chamber test: (**a**) total time spent in the social chamber (SC), center chamber (CC), and non-social chamber (NSC), indicating significant difference between time spent in SC vs. NSC amongst all groups. (**b**) Total number of entries into the social and non-social chambers among all groups, indicating significant difference among VPA males and VPA females. (**c**) Sociability index, indicating no significant difference between the males and females of both the VPA-exposed and the control groups. All data are presented as mean ± standard deviation. VPA: valproic acid. * *p* ≤ 0.05, ** *p* ≤ 0.01, *** *p* ≤ 0.001.

Time spent in each of the three chambers in the apparatus was quantified to determine any preference towards any of the chambers, i.e., social and non-social (Figure 2a).

For the center chamber, we found no significant difference in time spent between the animals exposed or unexposed to VPA [F(1, 28) = 3.24, *p* = 0.08] or between the sexes [F(1, 28) = 1.43, *p* = 0.24]. When examining the interaction of drug exposure and sex effect, we found no significance [F(1, 28) = 0.95, *p* = 0.34]. All the groups, whether male or female, exposed to VPA or not, showed the same level of interest in the center chamber.

When examining the time spent in the non-social chamber, no effects were noted related to drug exposure [χ^2^ (1) = 0.28, *p* = 0.60] or sex [χ^2^ (1) = 0.006, *p* = 0.94]. Valproic acid exposure did not alter the time spent in the non-social chamber for either males or females in the treated group.

Drug exposure had no effect on the duration spent in the social chamber [F(1, 28) = 1.24, *p* = 0.28], and nor did sex [F(1, 28) = 0.17, *p* = 0.68]. Assessing the sex and drug interaction, we found no significant difference among the groups [F(1, 28) = 3.99, *p* = 0.056]. All groups showed the same affinity for the social chamber regardless of sex or prenatal exposure to VPA.

We then assessed the relative affinity for the social vs non-social chamber in each group. The VPA-exposed males showed a greater preference for the social chamber over the non-social chamber [t7 = 5.67, *p* < 0.001], a pattern which was also observed in their female counterparts [t7 = 3.45, *p* = 0.01]. The same preference for the social chamber was evident in control males [t7 = 8.02, *p* < 0.001] and females [t7 = 8.83, *p* < 0.001]. Overall, time spent in the social vs. non-social chamber, in all the groups, whether male or female, exposed to VPA or not, showed the same increased affinity for the social chamber.

The number of entries into the social and non-social chambers were recorded and compared between the groups (Figure 2b). The results showed no significant differences in entry numbers to the non-social chamber between groups when classified by drug exposure [F(1, 28) = 0.05, *p* = 0.82], by sex [F(1, 28) = 0.16, *p* = 0.69], or by interaction between the two [F(1, 28) = 0.73, *p* = 0.4]. When considering the social chamber, the number of entries was also found to be similar when considered based on drug effect [F(1, 28) = 2.85, *p* = 0.1], sex effect [F(1, 28) = 2, *p* = 0.17], and on the two factors’ interaction [F(1, 28) = 1.22, *p* = 0.28].

The VPA-exposed males entered the social chamber significantly more than they did the non-social chamber [t7 = 2.72, *p* = 0.03], while the VPA-exposed females showed the same pattern of increased entry into the social chamber [t7 = 3.37, *p* = 0.01]. In the control group, the females showed no difference in their entry frequencies between the social and non-social chambers [t7 = 1.93, *p* = 0.10], a pattern observed also in the male cohort [t7 = 1.64, *p* = 0.14]. The VPA-exposed groups (both male and female) entered the social chamber more frequently than their control counterparts.

Sociability index: a sociability index was calculated for each group of animals and compared across drug exposure and sex. Drug exposure was found to have no influence on the sociability index [χ^2^ (1) = 0.28, *p* = 0.60], and nor did sex [χ^2^ (1) = 0.02, *p* = 0.88] (Figure 2c).

### 3.2. Valproic Acid Effects on Non-Verbal Communication

#### Cotton-Tip Based Olfactory Test

Habituation was defined as a significant decreasing pattern in rat interaction or sniffing time with repeated presentations of the same scent.

**Figure 3:**
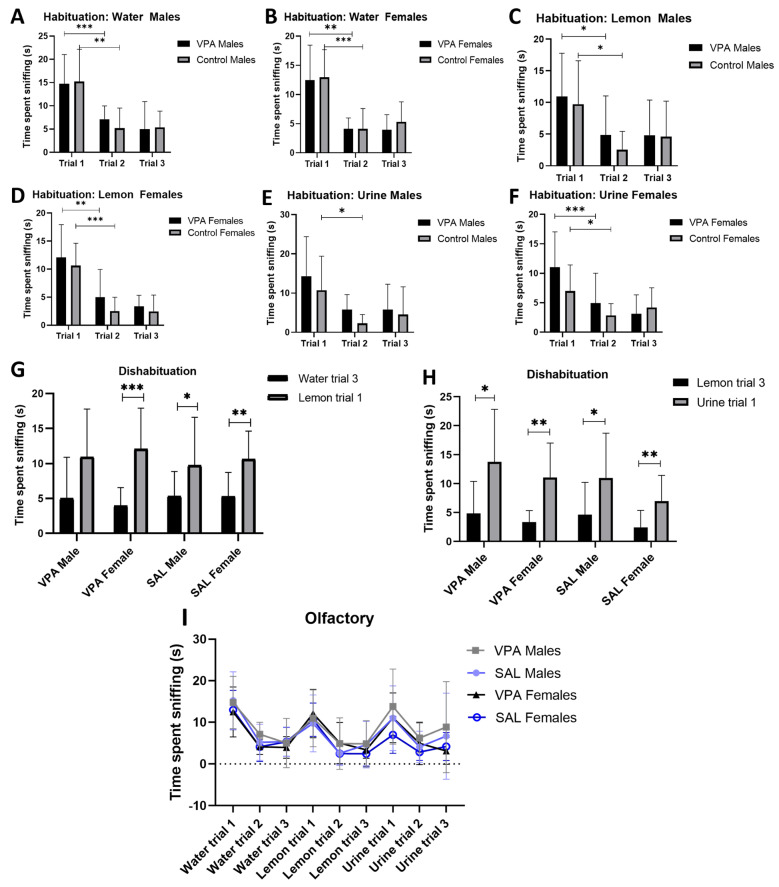
Non-verbal communication. Graphs showing outcomes from the cotton-tip test, total time spent sniffing (habituation): Time spent sniffing the water scent for males (**A**) and females (**B**). The time spent sniffing the lemon scent for males (**C**) and females (**D**). The time spent sniffing the urine scent by males (**E**) and by females (**F**). Outcomes for dishabituation: (**G**) shows the time spent sniffing familiar water scent (trial 3) followed by novel lemon scent (trial 1); (**H**) shows the time spent sniffing novel urine scent (trial 1) following familiar lemon scent (trial 3). Summarized habituation and dishabituation patterns for all groups (**I**). All data are presented as mean ± standard deviation. VPA: valproic acid, SAL: 0.9% saline solution. * *p* ≤ 0.05, ** *p* ≤ 0.01, *** *p* ≤ 0.001.

Regarding the time spent sniffing water (a neutral scent), all the animals managed to habituate to the water scent. We conducted a paired *t*-test comparing water trial 1 to water trial 2 and found that the VPA-exposed males showed a significant decrease in interaction with repeated presentations of water [t9 = 3.66, *p* = 0.005, Figure 3A,I], while their control counterparts showed a similar significantly decreasing pattern [t10 = 4.106, *p* = 0.002, Figure 3A,I]. The significant decreasing pattern upon repeated presentations continued to be apparent for females exposed to VPA [t10 = 4.502, *p* = 0.001, Figure 3B,I] and those not exposed [t11 = 6.456, *p* < 0.001, Figure 3B,I].

Regarding the lemon scent (a non-social scent), the VPA-exposed males were able to habituate as the repeated presentations of the odor showed a significantly decreasing trend [t9 = 2.837, *p* = 0.02, Figure 3C,I]. The control males also showed habituation to lemon scent [t10 = 5.413, *p* < 0.0003, Figure 3C,I]. Both the VPA-exposed [t10 = 4.36, *p* = 0.001, Figure 3D,I] and the control [t11 = 7.294, *p* < 0.0001, Figure 3D,I] females managed to habituate to the scent as well.

Finally, we presented opposite sex urine (a social scent) repeatedly to each group and observed that the VPA-exposed males habituated significantly [t9 = 2.522, *p* = 0.033, Figure 3E,I], and so did the control males upon repeated presentations [t10 = 2.937, *p* = 0.0149, Figure 3E,I]. For the females, the VPA-exposed group habituated successfully [t10 = 3.140, *p* = 0.011, Figure 3F,I], and so did the control females [t11 = 2.842, *p* = 0.016, Figure 3F,I]. Overall, all the groups habituated appropriately to all odors presented between trials 1 and 2 and did not change further with the presentation of trial 3.

We assessed the ability to recognize a novel scent following a familiar one, an indication of dishabituation, by comparing the interaction time between the third presentation of a familiar scent and the first presentation of a novel scent, i.e., from water to lemon, and from lemon to urine. The assessment revealed that VPA-exposed males failed to dishabituate when transitioning from water to lemon [t9 = 1.85, *p* = 0.1, Figure 3G,I], while the VPA-exposed females did manage to dishabituate [t10 = 6.05, *p* = 0.0001, Figure 3G,I]. In the control group, the males [t10 = 2.59, *p* = 0.03, Figure 3G,I] and females [t11 = 3.78, *p* = 0.003, Figure 3G,I] both successfully dishabituated from water to lemon.

When the initial presentation of urine followed the last presentation of lemon, the VPA-exposed males [t9 = 2.33, *p* = 0.04, Figure 3H,I] dishabituated, and so did the VPA-exposed females in the group [t10 = 4.5, *p* = 0.001, Figure 3H,I]. With respect to the control cohort, the males dishabituated [t10 = 2.46, *p* = 0.03, Figure 3H,I] and so did their female counterparts [t11 = 3.12, *p* = 0.01, Figure 3H,I].

Only the VPA-exposed males failed to dishabituate at one transition point (from neutral to non-social scents), while all other groups dishabituated successfully.

### 3.3. Valproic Acid Effects on Exploratory, Repetitive, and Anxiety-like Behaviors

#### 3.3.1. Assessing Exploratory Behavior

**Figure 4:**
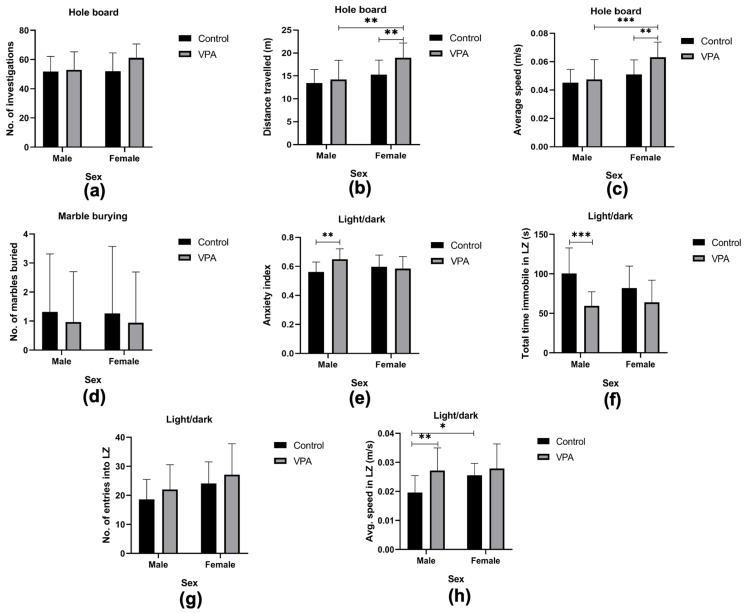
Repetitive/restrictive behavior, lack of interest and anxiety. Outcomes from the hole board test: total number of investigations (head dips into holes) in the hole board test, indicating no significant differences among the groups (**a**). Total distance travelled while undertaking the hole board test, indicating the VPA-exposed females travelling longer than their male counterparts, and significantly longer than their control female counterparts (**b**). (**c**) indicates the average speed of travel, where a significant difference between the females in the VPA-exposed and control groups was seen, along with a significant difference between the males and females of the VPA-exposed group. Graph showing the outcome from the marble-burying test: (**d**) number of marbles buried by each group, where no significant difference was found. Graphs showing the outcomes of the light/dark test: (**e**) shows the anxiety index, where a significant difference was found between the male groups. The total time spent immobile in the light zone indicated a significant difference within the male groups (**f**). (**g**) indicates the number of entries made into the light zone, indicating no significant difference between any groups. The average speed in the light zone revealed a significant difference between the males of the VPA-exposed and the control males (**h**), along with a significant difference between males and females within the control group. All data are presented as mean ± standard deviation. VPA—valproic acid, LZ—light zone. * *p* ≤ 0.05, ** *p* ≤ 0.01, *** *p* ≤ 0.001.

The hole board test simulates a novel environment and assesses the willingness to explore. This represents the human autism characteristic of lack of openness to explore the unknown. With regard to the number of investigations in the hole board apparatus, we observed no significant differences when compared in terms of drug exposure [F(1, 71) = 3.64, *p* = 0.06], sex [F(1, 71) = 2.47, *p* = 0.12] or interaction between sex and drug exposure [F(1, 71) = 2.31, *p* = 0.13]. No group appeared to explore the apparatus significantly more than any other in this test (Figure 4a).

The total distance travelled within the apparatus indicated a significant difference when drug exposure [F(1, 71) = 7.92, *p* = 0.006] and sex [F(1, 71) = 16.98, *p* < 0.001] were considered, but not the interaction between the two variables [F(1, 71) = 3.23, *p* = 0.08]. Post hoc comparisons showed that within the VPA-exposed group, the females travelled significantly more than the males (*p* = 0.01, Figure 4b), while the control males and females showed no differences in travel distance (*p* = 0.34, Figure 4b). The males across drug exposure were not statistically different from each other (*p* = 0.91, Figure 4b), while the VPA-exposed females travelled longer than the control females (*p* = 0.005, Figure 4b). The VPA-exposed females were found to have travelled greater distances than all other groups.

For average speed of travel within the hole board apparatus, differences were observed along drug exposure [F(1, 71) = 7.71, *p* = 0.007] and sex [F(1, 71) = 16.98, *p* < 0.001], but no differences were observed for the interaction between the two variables [F(1, 71) = 3.68, *p* = 0.06]. Post hoc comparisons revealed that across drug exposure, the VPA-exposed males exhibited no difference in speed of travel from the control males (*p* = 0.94, Figure 4c), while the VPA-exposed females travelled significantly faster than the control females (*p* = 0.004, Figure 4c). Comparing sex differences, the VPA-exposed females showed higher speeds than the males (*p* < 0.001, Figure 4c). We found no difference in speeds of travel between the two sexes (*p* = 0.35, Figure 4c) in the control groups. Taken together, the parameters assessed in the hole board test indicate that the VPA-exposed female group displayed hyperlocomotion.

#### 3.3.2. Assessing Repetitive Behavior

Following a 20 min period left uninterrupted in the marble burying apparatus, the marbles were photographed to note degree of concealment from the surface of the sawdust. The urge to bury marbles shows a tendency towards repetitive behavior, which is equivalent to the repetitive behavior associated with one of the symptoms of autism. There was no drug effect on the number of marbles buried between the controls and the VPA-exposed autism model [χ^2^ (1) = 0.42, *p* = 0.52]. In addition, sex played no role in the number of marbles buried [χ^2^ (1) = 0.49, *p* = 0.49, Figure 4d].

#### 3.3.3. Assessing Anxiety-like Behavior

To assess anxiety levels, we conducted the light/dark test and noted the relative time spent in the dark zone (expressed as anxiety index), total time immobile (freezing behavior), and speed of travel within the light zone, as well as the number of entries into the light zone.

From the time spent in each compartment, i.e., light vs dark, an anxiety index was calculated for each of the groups. Drug exposure did predispose animals to spending a greater length of time in the dark [F(1, 71) = 4.50, *p* = 0.04]. However, sex did not predispose groups to spending greater times in the dark zone [F(1, 71) = 0.75, *p* = 0.39]. It was found that being of a certain sex and exposed or not exposed to VPA was also a predisposing factor [F(1, 71) = 7.85, *p* = 0.007]. Post hoc comparisons among the exposed vs unexposed males showed that the VPA-exposed males spent significantly higher durations in the dark zone, resulting in a higher anxiety index than controls (*p* = 0.005, Figure 4e). When females were compared, there were no differences between the exposed group and their control counterparts (*p* = 0.96, Figure 4e). The VPA-exposed group had no differences when males and females were compared (*p* = 0.08, Figure 4e), nor did the control group between males and females (*p* = 0.46, Figure 4e). Only the VPA-exposed males were found to have spent significantly more time in the dark zone than other groups, an indication of their heightened anxiety-like behavior, confirmed by the higher anxiety index (Figure 4e).

The time that animals had spent immobile was quantified, and yielded an outcome where drug exposure was a predisposing factor for freezing time [F(1, 71) = 21.18, *p* < 0.0001]. There was no significant difference observed from sex effect [F(1, 71) = 1.17, *p* = 0.28], nor the interaction between drug exposure and sex effects [F(1, 71) = 3.17, *p* = 0.08]. Post hoc comparisons showed that the VPA-exposed males had spent significantly less time freezing when compared to control males (*p* = 0.0002, Figure 4f), while the VPA females showed no significant difference from the control females (*p* = 0.19, Figure 4f).

Exposure to VPA did not affect the number of entries [F(1, 71) = 2.71, *p* = 0.10] into the light zone, while sex did significantly alter the number of entries into the light [F(1, 71) = 7.53, *p* = 0.008]. Interaction between drug exposure and sex did not appear to affect the number of entries into the light zone [F(1, 71) = 0.01, *p* = 0.92]. Post hoc comparisons revealed no significant differences in any of the groups concerned (Figure 4g).

Average speed of travel while in the light zone: exposure to VPA did have an impact on the average speed of travel in the light zone [F(1, 71) = 10.64, *p* = 0.002], and so did sex [F(1, 71) = 4.81, *p* = 0.03]. However, when combining the two variables, their interaction yielded no significant impact [F(1, 71) = 3.03, *p* = 0.09]. Post hoc comparisons revealed that VPA-exposed males had travelled at higher speeds than their control counterparts (*p* = 0.005, Figure 4h), while the females (exposed and controls) showed similar speeds (*p* = 0.7, Figure 4h). In the VPA-exposed group, the males and females had comparable speeds of travel (*p* = 0.99, Figure 4h), while the males in the control group had travelled much slower than their female counterparts (*p* = 0.02, Figure 4h).

Overall, the VPA-exposed males travelled faster and spent more time in the dark zone (higher anxiety index), indicating their heightened level of anxiety-like behavior together with some hyperactivity.

#### 3.3.4. Assessment of Motor Abilities

**Figure 5:**
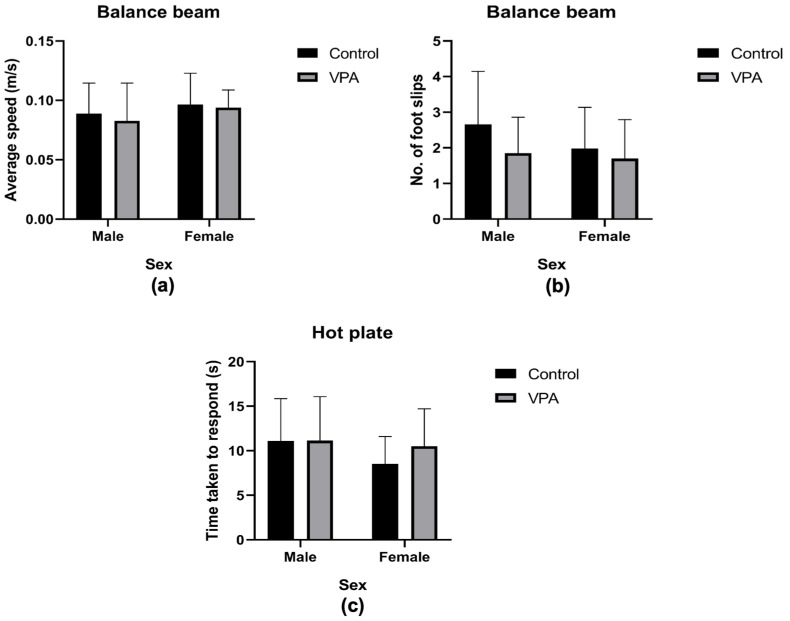
Motor and sensory stimuli. Graphs showing outcomes for the balance beam test: The average speed across the balance beam indicated non-significant differences between all groups (**a**). The number of foot slips was not significantly different between the groups (**b**). Graphs showing the outcomes for the hot plate thermal test: (**c**) shows the time taken to react to thermal stimuli, which indicated no significant difference between the groups. All data was presented as mean ± standard deviation. VPA—valproic acid.

In the balance beam test, there was no significant difference observed in average speed across the beam (m/s) between the control and VPA-exposed models [χ^2^ (1) = 0.51, *p* = 0.48, Figure 5a]. Comparing between the sexes, there was no significant difference found between the males and females in average speed of travel across the beam [χ^2^ (1) = 3.23, *p* = 0.07, Figure 5a].

There was no significant difference in number of foot slips (errors) when compared by drug exposure [F(1, 57) = 3.20, *p* = 0.08], nor by sex [F(1, 57) = 1.82, *p* = 0.18]. When considering the interaction between the drug exposure and sex, we also found no significant differences [F(1, 57) = 0.75, *p* = 0.39]. There were no significantly heightened errors made by any of the groups while traversing the beam (Figure 5b).

#### 3.3.5. Assessing Nociception

The hotplate test was used to assess the nociceptive threshold to a mildly painful thermal stimulus. Across drug exposure, no differences in the threshold were observed [χ^2^ (1) = 1.43, *p* = 0.23], and similar observations were made with regard to the sex effects [χ^2^ (1) = 2.28, *p* = 0.13, Figure 5c].

#### 3.3.6. Assessing Cognitive Abilities

**Figure 6:**
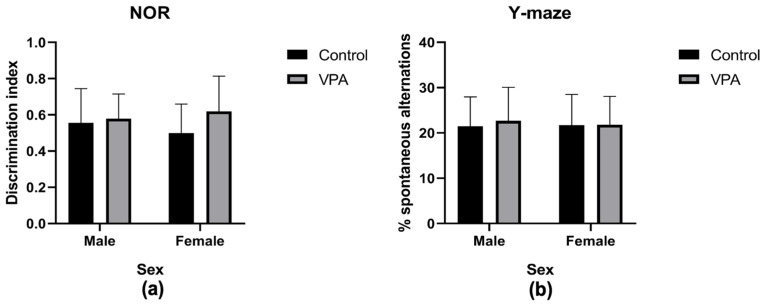
Cognition. Graph showing the outcomes for the novel-object recognition (NOR) test: (**a**) shows the discrimination index which revealed no significant difference between the four groups. Graph showing the outcome of the y-maze test: (**b**) shows the percentage of spontaneous alternations, indicating no significant difference between all groups. All data are presented as mean ± standard deviation. VPA—valproic acid.

Cognition was assessed using the novel object recognition test. After calculating the discrimination indices for the novel object, analyses showed that drug exposure had no impact on the ability to recognize the novel objects since the time spent with the familiar and novel objects was not significantly different [F(1, 70) = 3.10, *p* = 0.08]. Sex similarly did not impact the discriminative ability towards the novel object between the groups [F(1, 70) = 0.04, *p* = 0.85]. The interaction between sex and drug exposure also yielded no significant impact upon this parameter [F(1, 70) = 1.4, *p* = 0.24]. This assessment revealed no impaired discriminative abilities in the VPA exposed group of either sex (Figure 6a) since the indices were all above 0.5 (or 50%), indicating a clear recognition of the novel object in all groups.

#### 3.3.7. Assessing Spatial Memory and Repetitive Behavior

No differences were observed when assessing the level of spontaneous alternations by drug exposure [F(1, 71) = 0.18, *p* = 0.68], sex [F(1, 71) = 0.04, *p* = 0.84], or by sex and drug exposure interaction [F(1, 71) = 0.12, *p* = 0.73]. The Y-maze test revealed the same levels of spatial short-term memory and no heightened repetitive behaviors in any group (Figure 6b).

## 4. Discussion

This study aimed to expand the descriptions of behavioral features in valproic acid-induced male and female rat models of autism, as most research has focused only on males [28,29,51]. Behavioral tests revealed various autism-like phenotypes in both sexes, confirming that prenatal exposure to valproic acid induces such behaviors, including altered social communication, hyperactivity, and increased anxiety. These effects varied between males and females. The behavioral repertoire observed was reminiscent of previous findings [18,52], but also showed some mixed results. Some expected outcomes were not evident in the current study, such as impaired sociability from the three-chamber test, hypoalgesia from the hot-plate test, cognitive impairment from the Y-maze and novel recognition test, motor impairments from the balance beam test, and repetitive behavior from the hole-board test. The mixed nature of outcomes supports a recommendation that future studies should include a broader range of behavioral studies in each symptom domain. In addition, a more useful approach would be to first optimize the VPA dosage and exposure time window ahead of each study, as different environmental factors may be confounding outcomes and limiting the reproducibility of previous outcomes from studies conducted elsewhere.

- Prenatal valproic acid exposure leads to impaired sociability

In the three-chamber sociability test, VPA-exposed rats (both male and female) showed no social impairments and performed similarly to non-exposed rats, displaying a strong preference for the social chamber. This was unexpected, especially for VPA-exposed males, as previous studies often reported social interaction impairments in this group [18,29,53]. However, in line with the current one, some studies with similar methodologies have also found no impairments [18,54]. The protocols for creating the VPA model of autism vary between studies in terms of VPA concentration and timing of exposure, which may explain the mixed sociability outcomes. Previous studies have used concentrations ranging from 350 to 600 mg/kg [24,53,55] and varied exposure times from gestational days GD9.5 to GD15 [27,30,55]. Our study used a common method, injecting 600 mg/kg of VPA subcutaneously on gestational day (GD) 13. Kim et al. [27] previously found that social impairment in VPA-exposed rats varies with the timing of exposure, with earlier injections (GD7 and GD9.5) having no effect, and effects peaking at GD12, then declining rapidly thereafter towards GD15. Our study’s lack of impairment might be due to injections at GD13, which is after the peak window. Although GD12 and GD13 are close, it appears that even a day’s difference can affect outcomes, explaining our findings. This further supports Kim et al.’s assertion that the timing of VPA exposure is crucial in determining the severity of social impairments. Although most practical, the use of a single exposure period and dosage presents a limitation in the current study as well as others similar to it, given the variability of behavioral outcomes that result from subtle changes in these aspects. To mitigate such limitations to this promising model, studies can conduct a pilot phase to identify the ideal exposure window and dosage ahead of main investigations, allowing for the optimal representation of ASD in the model for maximum learning opportunities therefrom.

Chaliha and colleagues [56] identified four key evaluations for social behavior in VPA-induced rodent autism models: ultrasonic vocalization, social preference, social novelty preference, and social interaction. Our study focused on social interaction, comparing preference between an inanimate object and a conspecific animal. Jabarin and colleagues [57] highlighted the limitations of the three-chamber sociability test, noting it can yield mixed results due to factors like acclimatization length, age at testing, and specific social stimuli. They argue the test is somewhat simplistic and may miss some social impairments. Assessing all four sociability paradigms might have revealed social impairments in our VPA-exposed group, overcoming these limitations. It was important in this study to limit the test to one evaluable paradigm in efforts to minimize stress. A larger study design using a new sample for each of the four paradigms may be of use when conducting this test.

The DSM-V [6] outlines autism diagnosis criteria, including deficits in nonverbal social communication. Our VPA-exposed males showed impaired olfactory dishabituation, reflecting this core ASD deficit. Markram et al. [58] found similar social deficits in VPA models, supporting our findings. Mabunga et al. [59] argued that similarly, communication should be part of sociability observations in animal models, just as they are now included in the DSM-V. Although ultrasound vocalization was not investigated herein, impaired olfaction in VPA-exposed male rats serves as a proxy for impaired human non-verbal communication [35,37,60], providing evidence for communication impairment in the VPA-exposed males of the current study.

Valproic acid-exposed males showed impaired dishabituation, which may indicate a communication deficit beyond simple olfactory function, reflecting social communication issues [33]. Further support for this interpretation is offered by Belzung and Lemoine [61], who emphasize the importance of behavior meaning over material similarity in animal models. Crawley [33] notes that while rodent olfaction differs anatomically from human language and visual communication, the behavioral outcomes of impaired communication are similar. Thus, rat olfactory function approximates non-verbal communication in humans [18,52,54], which is often impaired in autism, and can be quantified similarly [33]. The failure to dishabituate in the present study’s VPA male rats suggests an impaired ability to register new messages, possibly due to reduced motivation to explore new scents and difficulty discerning social stimuli [37,62,63], coupled with an inability to discern salient from non-salient social stimuli [33,37]. Many authors [35,37,60] suggest this impaired dishabituation to be a sign of compromised non-verbal communication. The current VPA-exposed males showed the core ASD diagnostic feature of impaired social abilities.

- Prenatal valproic acid exposure leads to hyperactivity but not impaired exploration

Another key finding of the present study was that VPA-exposed animals exhibited heightened levels of locomotion while undergoing various tests. Increased locomotion is a well-established indication of hyperactivity [18,64,65], equivalent to the various hyperactive behaviors seen in autism. Both males and females exposed to VPA exhibited a tendency towards hyperactivity in the three-chamber test, while the females were additionally hyperactive in the hole board test, and the males in the light/dark test. Surprisingly, VPA exposure did not significantly alter head-dipping behavior in the hole board test for either males or females, contrasting with previous studies that reported reduced exploration in VPA-exposed animals [30,51]. The heightened locomotion levels observed in VPA-exposed females, in the absence of increased head dipping, suggest hyperactivity rather than increased exploratory efforts.

Notably, hyperlocomotion was observed equally in VPA-exposed males and females, offering additional information, as previous studies have tended to investigate males only [30,50]. Valproic acid-exposed males also showed less freezing behavior in the light/dark test, indicating hyperactivity. Shaywitz et al. [66] have linked hyperactivity to catecholaminergic system dysfunction, particularly in the dopamine and norepinephrine systems, a finding confirmed by Choi et al. [67]. Valproic acid exposure likely causes epigenetic changes in these systems, disrupting dopamine and norepinephrine homeostasis and leading to hyperactivity seen in both sexes of the current study.

- Motor, sensory, cognitive, and repetitive behavior impairments were not apparent after valproic acid exposure

This study found unexpected results regarding motor, sensory, cognitive, and repetitive behaviors from the balance beam, Y-maze, marble burying, and novel object recognition tests, which were all unimpaired by VPA exposure. Although VPA-exposed females completed the balance beam task faster than males, the difference was not statistically significant, and all groups performed similarly. This contrasts with Main and Kulesza [68], who reported impaired motor responses in VPA-exposed animals and linked this to reduced cerebellar Purkinje cell size. Our findings suggest that the cerebellar Purkinje cells in our treated animals might not have been significantly impacted; however, no analysis of cerebellar Purkinje cells was undertaken to confirm this, limiting the extent to which we can make conclusions surrounding this aspect. It is also possible that the balance beam test was not sensitive enough to detect subtle motor impairments.

Like motor output, the sensory response in the VPA-exposed groups of both sexes remained unimpaired when subjected to a nociceptive stimulus. This contradicts Kerr et al. [65], who reported hypoalgesia to heat in VPA-exposed rats, and the DSM-V [6], which notes hypoalgesia among autism endophenotypes. Our findings align with Fereshetyan et al. [69], showing a heterogeneous record for this endophenotype. Kerr and colleagues [70] linked altered endocannabinoid systems in VPA-exposed rats to hypoalgesia, while Militerni and colleagues [71] found a correlation between hypoalgesia and elevated serotonemia in autistic children. Investigating the endocannabinoid system and serotonin level changes in VPA-exposed animals could clarify their roles in autism-related reduced pain perception.

The second core diagnostic feature of autism, alongside social impairment, is repetitive, restrictive patterns of behavior [6]. We used marble burying and Y-maze tests but found no significant repetitive behaviors in any group. Nicolini and Fahnestock [72] and Kim et al. [73] noted that prenatal VPA exposure alters acetylcholine levels, with donepezil (an acetylcholinesterase inhibitor) reversing repetitive behaviors. This was supported by Friedman et al. [74] and Karvat and Kimchi [75], who found elevated acetylcholinesterase levels linked to repetitive behaviors. Such important information is based on the ability to observe clear repetitive behaviors in animal models. Studies have offered insight on why negative results can arise in this assessment including task complexity [76], variability in VPA dosage and timing [56], sex differences [77], and genetic and neurobiological factors [78,79]. All of this highlights the complexity of studying autism-like behaviors in animal models.

This study found no cognitive impairments in VPA-exposed rats compared to unexposed ones using the novel object recognition and Y-maze tests. Cognitive test outcomes after VPA exposure vary widely [56], with some studies finding no differences [28,29,31,80] and others reporting impairments [29,81]. Rendall et al. [76] suggested that task complexity may affect cognitive impairment detection, with simpler tasks sometimes missing impairments and complex tasks revealing them more readily. This effect is also seen in human assessments [82]. Another reason for this variability is the fact that autism is not always accompanied by cognitive impairments [6], and when present, they are highly variable [6].

- Prenatal valproic acid exposure increased anxiety-like behaviors

Our study found increased anxiety-like behavior in VPA-exposed males during the light/dark test, consistent with previous findings [83,84], which indicates inappropriate fear conditioning in the group. Anxiety, though not a core autism symptom, is a common comorbidity [72]. South et al. [85] reported a positive correlation between sociability and fear conditioning in autistic individuals. Having found both heightened anxiety-like behaviors and impaired sociability in our VPA-exposed males, our outcomes are consistent with South et al.’s [85] findings that abnormal fear conditioning leads to anxiety and impaired sociability. This implies that the ability to learn appropriate fear responses is correlated to the ability to form appropriate social interactions. Further research on improving fear conditioning in VPA-exposed rat models could help enhance interventions that seek to improve social interaction skills, which could then be generalized to humans. In the brain, the amygdala has been identified as a key area in processing fear responses and thus underlying much of the heightened anxiety seen in autistic individuals [58]. Mechanisms reportedly include hyperreactivity to stimulation, diminished inhibition, and hyperplasticity in the amygdala of VPA-exposed offspring [58]. Although not examined directly in the current work, it seems reasonable to suspect these features in the male VPA group’s amygdalae.

- Sex differences in behavioral outcomes following valproic acid exposure

The VPA-exposed males in the current study were the only group to show some social impairment with respect to the domain of non-verbal communication, which was consistent with findings of a large review of similar studies by Chaliha et al. [56]. They asserted that females likely have some compensatory mechanism that protects them from social impairment following VPA exposure. In our study, VPA led to maladaptive learning of fear in males while females were spared, indicating that males and females potentially have different neurobiological responses to VPA exposure. For example, Kazlauskas et al. [77] found higher baseline plasma corticosterone levels in treated males, while treated females had levels similar to controls. Under stress, the treated males showed blunted corticosterone production, whereas females had heightened responses, indicating a more responsive hypothalamus-pituitary-adrenal (HPA) function in females [77]. Additionally, treated females had reduced astroglial and microglial (important for immunity) cell density in the dentate gyri and cerebelli compared to males [77]. Autistic boys also reportedly have more activated microglia in various brain regions including the cerebellum, relative to autistic girls [12]. Research in both animals and humans increasingly highlights prenatal immune disturbance as key in the onset of ASD, for example maternal immune activation [86,87]. Furthermore, once exposed to such predisposing environments for ASD, males and females respond differently, as evinced by the male bias of ASD outcomes [86]. The recent work of Arenella [86] reports clear correlations between the occurrence of autism and certain immune disorders, and indicates that the mechanisms underlying this sex-based discrepancy in vulnerability to ASD may lie in the immunology and immunogenetics of either sex [86]. They suggest that the same genetic variations leading to certain immune disorders are responsible for certain autistic traits. Interestingly, immune disorders have a sex-based bias of occurrence, just as autism does [86,88]. Sex hormones are likely at the center of why males’ and females’ immune systems behave differently [88], thus predisposing males to more ASD and few allergic immune disorders, but females to less ASD and more autoimmune disorders [86]. According to Roved et al. [88], testosterone effects immunosuppression while estrogen effects immunoenhancement, both leading the immune system to respond differently in each sex in the wake of immune challenges, for example maternal immune activation. Such neurological, physiological, and immunogenetic differences offer key insights as to why females may have a resilience mechanism against behavioral impairments typically triggered by VPA exposure (which simulates maternal immune activation [86]).

Genetic differences between males and females further support the theorized differences in autism vulnerability. The X-linked imprinting theory suggests that imprinted X-linked genes protect against autism traits, making males more vulnerable due to having only one X chromosome [78]. The empathizing-systemizing theory [79] posits that females’ empathy helps them form socially appropriate behaviors. These theories align with our study’s findings, where VPA-induced autism-like behaviors were more pronounced in males.

- The validity of the valproic acid model of autism

Ellenbroek and Cools [89] state that construct validity is the highest form of model validity. Our study animals have construct validity, based on established studies [18,52]. Despite mixed outcomes in cognition, motor abilities, and lack of overt repetitive behaviors (especially in VPA males), the model’s face validity holds when considering impaired non-verbal communication, hyperactivity, and heightened anxiety-like behavior. The prior literature supports the construct, face, and predictive validity of this model [56,59]. Previous empirical validation of molecular changes gives further evidence of the model’s validity, such as oxidative stress, histone deacetylase inhibition, excitatory/inhibitory imbalance, and hyperserotonemia [59,73,90,91,92,93]. Given that both “core symptoms” of ASD were not observed, the current model lends itself to a limitation of questionable robustness, which molecular investigations could have resolved. As such, we recommend the coupling of such techniques in future similar studies.

Mabunga et al. [59] and Jabarin et al. [57] suggest that face validity can be influenced by various factors affecting behavior in specific contexts. The VPA autism model’s face validity should include more than just social impairment and repetitive behaviors. Our model showed ASD-like behaviors, with hyperactivity in both sexes and additional autism-like features in males, including social impairment and anxiety-like behaviors. These findings align with typical descriptions of human male vs female autism, where females seem genetically or neurobiologically protected from core autism signs.

## 5. Conclusions

This study adds evidence to the prenatal VPA rodent model of autism to enhance its validity and supports the establishment of a more standardized battery of behavioral tests to confirm autism-like phenotypes, along with the inclusion of both males and females in such studies. Further, the study highlights the importance of determining an exact ideal window and dosage for VPA exposure in creating the most representative models. As such, we recommend the piloting of varying exposure periods and dosages prior to conducting every study, such that the ideal exposure period can first be established for the specific environment. Both males and females in the current study exhibited altered behavioral traits consistent with core or associated autism features respectively. Males were the only group to show a core symptom of autism together with comorbidities, while females only exhibited a comorbidity and no core symptoms. This study found these altered behaviors to be clearly divergent by sex, likely due to sex-specific physiological differences. As sex has been shown herein to be a real factor in behavioral outcomes, we recommend the inclusion of female animals in all studies modeling autism by VPA exposure or other reagents.

## Data Availability

The raw data supporting the conclusions of this article will be made available by the authors on request due to it being part of a larger study whose data is still being analyzed for other manuscripts’ publication.
